# Aqueous Extract of* Clerodendranthus spicatus* Exerts Protective Effect on UV-Induced Photoaged Mice Skin

**DOI:** 10.1155/2016/9623957

**Published:** 2016-10-25

**Authors:** Lan Wang, Xie Zhang, Yong-xian Li, Lie-qiang Xu, Cai-lan Li, Zhen-biao Zhang, Jia-li Liang, Zi-ren Su, Hui-fang Zeng, Yu-cui Li

**Affiliations:** ^1^The First Affiliated Hospital of Chinese Medicine, Guangzhou University of Chinese Medicine, 12 Airport Road, Bai Yun District, Guangzhou 510405, China; ^2^School of Chinese Medicine, Faculty of Science, The Chinese University of Hong Kong, Shatin, Hong Kong; ^3^School of Chinese Materia Medica, Guangzhou University of Chinese Medicine, Guangzhou Higher Education Mega Center, 232 Wai Huan Dong Road, Guangzhou 510006, China

## Abstract

*Clerodendranthus spicatus *(Thunb.) C.Y.Wu (CS) is commonly used to treat kidney diseases in traditional Chinese medicine for its prominent anti-inflammatory effect and nourishing function to kidneys. In this study, aqueous extract of CS was assessed for its protective effect on UV-induced skin damage of mice. The chemical compositions of CS aqueous extract were determined by HPLC-ESI-MS/MS, in which 10 components were identified. During the experimental period, CS (0.9, 1.8, and 3.6 g/mL) was externally applied to shaved dorsal skins of mice prior to UV irradiation, daily for ten weeks. The results presented that CS (3.6 g/mL) apparently improved photodamaged skin appearance such as erythema, edema, and coarseness. The abnormal epidermal thickening was significantly reduced, and the dermal structures became more complete. The underlying protective mechanisms were associated with improving antioxidant enzymes activities including superoxide dismutase (SOD), catalase (CAT), and glutathione peroxidase (GSH-Px), downregulating inflammatory cytokines (IL-1*β*, IL-6, TNF-*α*, COX-2, and PGE_2_) expressions, recovering collagen density, and reducing matrix metalloproteinases productions. Sun protection factor of CS (3.6 g/mL) was 16.21 ± 0.03. Our findings for the first time demonstrated that CS had therapeutic effect on the photoaged skin. The results indicated that CS is a potential agent for photoprotective cosmetics.

## 1. Introduction

Long-term exposure to sunlight usually accelerates exogenous aging of skin, namely, photoaging. The characters of photoaging are laxity, coarse winkles, roughness, and irregular pigmentation [1]. Ultraviolet (UV) irradiation is the most important factor in the process of photoaging. It activates a complex cascade of biochemical reactions involving oxidative stress and inflammatory reaction and subsequently alters the structure and function of extracellular matrix (ECM) by degrading collagen and elastic fibers, finally leading to abnormal morphological and histological changes in human skin [2].

UV includes UVA, UVB, and UVC, with wavelengths of 320~400 nm, 280~320 nm, and 100~280 nm, respectively. Among them, both UVB and UVA can cause skin damage and photoaging [3]. UVB, mostly absorbed in the epidermis, mainly triggers proinflammatory mediators and stimulates the expression of matrix metalloproteinases (MMPs), which degrades ECM and ultimately results in wrinkles and sagging appearance [4]. Differing from UVB, UVA reaches the dermis. It generates reactive oxygen species (ROS), induces MMPs synthesis, and causes mutation of mitochondrial DNA. Therefore, on one hand, excess UV light could inhibit antioxidant enzymes activities, destroy the antioxidant defense systems, and finally bring about oxidative damage in the skin [1]. UV-induced oxidative stress further activates MAPK signaling pathway and then induces AP-1, resulting in increased activation of cytokines such as interleukins and tumor necrosis factor-*α* (TNF-*α*) [5]. On the other hand, UV expedites the production of NF-*κ*B and stimulates downstream inflammatory mediators such as interleukin-1*β* (IL-1*β*), IL-6, and TNF-*α*. These inflammatory mediators synergistically promote the accumulation of ROS, further augment the expression of MMPs, finally lead to erythema and edema, which are characters of inflammation responses. What is more, UV also stimulates skin cells to produce cyclooxygenase-2 (COX-2), catalyzing the synthesis of prostaglandin E2 (PGE_2_) from prostanoid precursors and deteriorating the skin inflammation [6].

To summarize, the excessive production of ROS and inflammatory cytokines is closely linked to the expression of MMPs and consequently causes coarse and corrugated skin. Upon the background of photoaging, we can speculate that antioxidant and anti-inflammatory agents have potentiality to delay this premature process [2].

*Clerodendranthus spicatus *(Thunb.) C.Y.Wu (*Orthosiphon stamineus*) (CS) is widely cultivated in tropical regions and the Pacific regions such as China, India, Malaysia, and Australia. It is a kind of traditional Chinese medicine to treat nephritis, lithangiuria, and cystitis [7, 8]. Based on the plenty researches we found CS contains various bioactive components such as flavonoids, diterpenes, and phenols [9] to exhibit the anti-inflammatory [10], analgesic [11], lipid peroxidation inhibiting [12], and free radical scavenging [13] activities, which are in agreement with mainstream therapies of containment of the progress of senescence [14]. What is more, in traditional Chinese medicine theory, kidney supports human body to be young and vigorous. Our team [15] and other researchers [16] have reported that CS has good antiaging effect through improving antioxidant capacity of aged mice induced by D-galactose. It attracts our attention to reveal if CS can prevent exogenous senescence. Therefore, this study aimed to investigate whether CS could remedy UV-induced photoaging.

## 2. Methods and Materials

### 2.1. Materials and Chemicals

We used CAT, SOD, GSH-Px, MDA, and mouse hydroxyproline (Hyp) assay kits (Jiancheng Institute of Biotechnology, Nanjing, China), as well as ELISA kits: TNF-*α*, IL-6, MMP-1, MMP-3, IL-1*β*, COX-2 and PGE_2_ (Cheng Lin Biological Technology Co., Ltd., Beijing, China). All other reagents used were of analytical grade.

### 2.2. Preparation of CS Aqueous Extract

CS (lot 20141101) was gifted by XinXing Tongren Pharmaceutical Co., Ltd. (Yunfu, China). 1 kg of dried plants added to 12-fold distilled water was soaked overnight. After being decocted for 2 h twice, the extracted solution was merged and concentrated to 1 L and then mixed with 2 L 95% ethanol for 24 hours. After that, the solution was filtered and the ethanol was recycled by rotary evaporator. The filtered fluid was diluted by distilled water to 0.1 L (10 g crude drug/mL) and then yielded to three different concentrations: 0.9 g/mL, 1.8 g/mL, and 3.6 g/mL.

### 2.3. HPLC-ESI-MS/MS Analysis of CS Aqueous Extract

HPLC analysis was executed on a Shimadzu LC-20A HPLC system consisting of a SPD-M20A PDA detector, a LC-20AD pump, a SIL-20AC automatic sampler, and CTO-20A thermostatic column compartment (Shimadzu Kyoto, Japan). A Kromasil KR100-5C18 column (250 mm × 4.6 mm, E17096) whose flow rate is 0.4 mL/min, column temperature is 25°C, and injection volume is 10 *μ*L was used for separation. CS was diluted to the concentration of 1 mg/mL with methanol and filtered through 0.22 *μ*m microporous membrane for HPLC-electrospray ionization-MS (HPLC-ESI-MS/MS) analysis. The mobile phase included water (solvent A) and acetonitrile (solvent B) and was set in gradient mode (0–10 min: 5%B→8%B; 10–15 min: 8%B→18%B; 15–25 min: 18%B; 25–40 min: 18%B→30%B; 40–50 min: 30%B→35%B; 50–52 min: 35%B→40%B; 52–57 min: 40%B→45%B; 57–67 min: 45%B→90%B; and 67–77 min: 90%B).

ESI source for mass detection was performed via Triple™ TOF 5600 system. Data acquisition and process were conducted with MultiQuant™ Software system (AB SCIEX, USA). Both positive and negative ionization modes of mass spectra were acquired. The optional parameters were set as follows: ion spray voltage (−4500 kV); ion source heater (550°C); curtain gas (N_2_, 35 psi); ion source gas 1 (55 psi); and ion source gas 2 (55 psi). The mass analyzer scan was 100 to 1000* m/z*. The entrance potential (EP) and declustering potential (DP) were −10 eV and −100 eV.

### 2.4. Animals

Female Kunming mice (6–8 weeks old, 18–22 g) were brought from animal center of Guangzhou University of Chinese Medicine (GZUCM, Guangzhou, China). All experiments were performed in specific pathogen-free lab and approved by the Institutional Animal Care and Use Committee at GZUCM (approval number SCXK (Guangzhou) 2014-0085). After acclimating for 1 week, mice were randomly divided into seven groups (*n* = 9). The grouping was as follows: naïve control (NC), sham control (SC), model control (MC), vehicle control (VC), and three treated groups (CS-low dose, CS-L; CS-middle dose, CS-M; and CS-high dose, CS-H). Experimenters anesthetized mice via inhaling ether and then used shavers to shave mice dorsal skins about 2.5 × 3 cm^2^. Except for NC group, all others were shaved. Hence the shaving was performed daily.

### 2.5. Establishing Photoaged Model and Treatment

The spectrum of UV light and ratio of UVB/UVA were set according to solar irradiation. Combined UVB and UVA lamps (Waldmann UV800, Germany) emitted the UV light. To control the UV radiation energy, an electronic controller was prepared. The UV lamp was fixed 30 cm above mice cages. The radiation energy supplied from the lamps (630 × 10 *μ*W) was measured with a UV radiometer (Waldmann Lichttechnik GmbH, Germany). The starting intensity of UV light in the first week was at one minimal erythemal dose (MED), which equaled 100 mJ/cm^2^. The following dose was increased by one MED every week until the fourth week. Then, the intensity of irradiation was at 4 MED for the remaining weeks [17]. The UV irradiation frequency was set at five times a week for ten weeks. Except for NC and SC group, all others groups were irradiated. Prior to being irradiated, the treated groups were externally embrocated with CS (0.9, 1.8, and 3.6 g/mL) and simultaneously the VC group were externally embrocated with distilled water every day during the whole experimental period.

### 2.6. Images Records of Dorsal Skins

Experimenters took photos of every dopey mouse skin weekly for 10 w. The macroscopic visual scores were calculated by blinded investigators according to the grading scale [18] (Table 1).

### 2.7. Histopathology Studies

The mice were sacrificed 24 h after the last CS treatment. The representative skin specimens were peeled and fixed in 10% formalin neutral buffered solution at least for 1 d to process paraffin sections. 5 *μ*m thick sections were stained with Hematoxylin & Eosin (H&E) for routine histology study. Besides, the sections were stained with Weigert's resorcin fuchsin to represent elastic fibers and Masson's trichromatic staining was used to reflect the density of collagen fibers [19].

In slides of every H&E stained section (magnification, 200x) hyperplastic epidermal layer was vertically measured at 10 selected locations using an optical microscope (Leica DMLB).

### 2.8. Measurements of Antioxidant Enzymes and Lipid Peroxidation in the Skin

Sample preparation was according to corresponding diagnostic kits. The skin tissue was homogenized with Ultra Turrax (T18 Basic, IKA) at 10,000 rpm for 20 seconds in 9-fold volumes of cold normal saline to get the 10% homogenate, 0.15 mL of which was taken out for the MDA assay. The remainder was centrifuged at 3,000 rpm for 20 min at 4°C to obtain supernatant used for CAT, SOD, GSH-Px, and protein concentration measurements.

### 2.9. Determination of Inflammatory Factors and MMP-1 and MMP-3 in the Skin

Another skin tissue was processed in the same way but in cold phosphate buffer solution and then the total supernatant was applied for IL-1*β*, IL-6, and TNF-*α*, COX-2 and PGE_2_, and MMP-1, MMP-3, and protein concentration.

### 2.10. Determination of Total Collagen Content

Collagen content can be converted from Hyp content, which was regarded as a characteristic amino acid of collagen, by multiplying the factor 7.46 [20]. About 100 mg fresh skin tissues were hydrolyzed in 6.0 M hydrogen chloride for Hyp kits to reveal the total collagen con.

### 2.11. Analysis of UV Absorption Spectrum and Sun Protection Factor (SPF)

CS aqueous extract was diluted to the concentration of 2 mg/mL with ethanol and filtered through 0.45 *μ*m microporous membrane for use. Full wavelength was set from 200 nm to 800 nm for analyzing the UV absorption spectrum. SPF is an important indicator to assess the photoprotection effect of sunscreen [21]. Ultraviolet spectrophotometry was used to determine SPF. The calculation was based on formulation (1) [22]. Optimized range to obtain absorbance was from 290 nm to 320 nm. Three doses of CS were, respectively, diluted 500 times with ethanol and filtered through 0.45 *μ*m microporous membrane for determination. Determinations were performed every 5 nm, repeated for 3 times at each point. The ultraviolet spectrometry photometer (Techcomp Ltd., UV1000) and matching 1 cm quartz cuvettes were used in the above detections:

(1) $$\begin{array}{c} SPF_{spectrophotometric}=CF\times \overset{320}{\underset{290}{\sum }}EE\left(\;ℷ\;\right)\times I\left(\;ℷ\;\right)\times Abs\left(\;ℷ\;\right). \end{array}$$

EE (*ℷ*): erythemal effect spectrum; I (*ℷ*): solar intensity spectrum; Abs (*ℷ*): absorbance of sunscreen product; and CF: correction factor (=10). The values of EE × I were constants determined by Sayre et al. [23].

### 2.12. Statistical Analysis

One-way analysis of variance (ANOVA) was used to detect group differences. A value of *p* < 0.05 was considered to be statistically significant. All analyses were performed using SPSS 17.0.

## 3. Results

### 3.1. Chemical Compositions of CS

**Figure 1:**
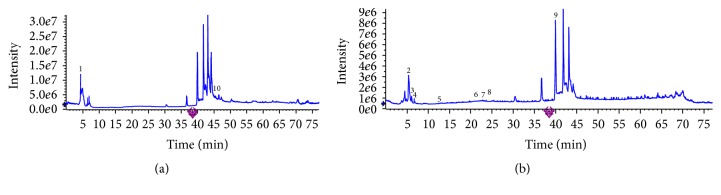
Total ion chromatorgraphies of CS aqueous extract by HPLC-ESI-MS/MS analysis. (a) Positive ion mode. (b) Negative ion mode.

The chemical compositions of CS aqueous extract were determined by HPLC-ESI-MS analysis, 10 components of which were identified as methyl rosmarinate, ethyl 3,4-dihydroxyphenyllactate, baicalein, danshensu, protocatechuic acid, p-hydroxybenzoic acid, rosmarinic acid, caffeic acid, ferulic acid, and 2*α*-hydroxy-ursolic acid presented in Table 2 and Figure 1.

### 3.2. CS Alleviated UV-Induced Skin Lesions

**Figure 2:**
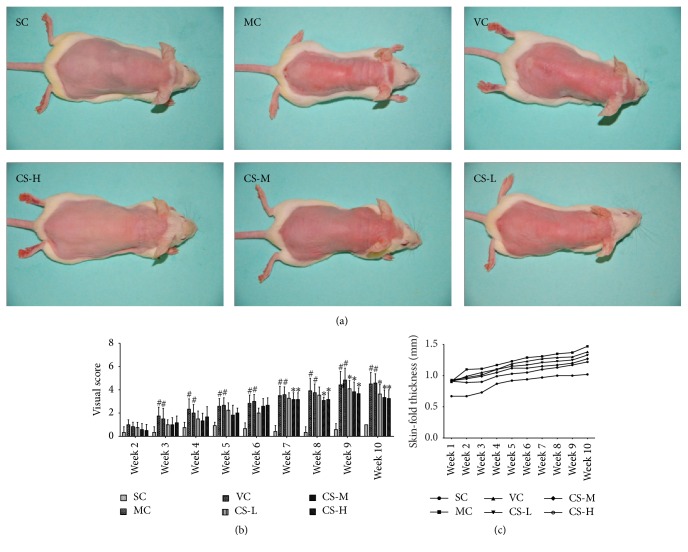
CS alleviated UV-induced skin lesions. (a) Visual appearance of different groups after the last treatment. (b) Visual scores of different treated groups. Data shown are the mean values ± SD (*n* = 9). ^#^ *p* < 0.05 compared with the SC group; ^*∗*^ *p* < 0.05 compared with the VC group. (c) Skin-fold thickness of different groups. Data shown are the mean values (*n* = 9).

The representative photos of UV-induced skin lesions were shown in Figure 2(a). After ten weeks, dorsal skins of mice in the MC and VC groups presented a coarse and corrugated appearance. Additionally, erythema, edema, and skin burns appeared frequently in these two groups. The abnormally changed skin appearances proved that the photoaging model in rats was successfully established, and the vehicle had no effect to ameliorate the UV-induced lesions. Contrarily, mice in the SC group which had not been irradiated showed neither winkles nor lesions, proving that the shaving operation had no macroscopic damage to skin. Compared to the VC group, the mice skin in the CS-H group presented much more smooth appearance without observable lesions. Meanwhile, dorsal skins of mice in the CS-M group displayed a few wrinkles, whereas mice in the CS-L group exhibited shallow wrinkles and slight erythema.

Statistically, the visual scores of SC group were much lower than that in both MC and VC groups from the third week (Figure 2(b)). However, after seven weeks, the visual scores of mice skin in CS-H and CS-M groups were obviously decreased (both *p* < 0.05, versus VC group), while the formation of wrinkles from the ninth week started to slow down in the CS-L group. These results indicated that topical application of CS could cure the UV-induced erythema and edema in a dose-dependent manner.

### 3.3. CS Reduced Epidermal Thickness

**Figure 3:**
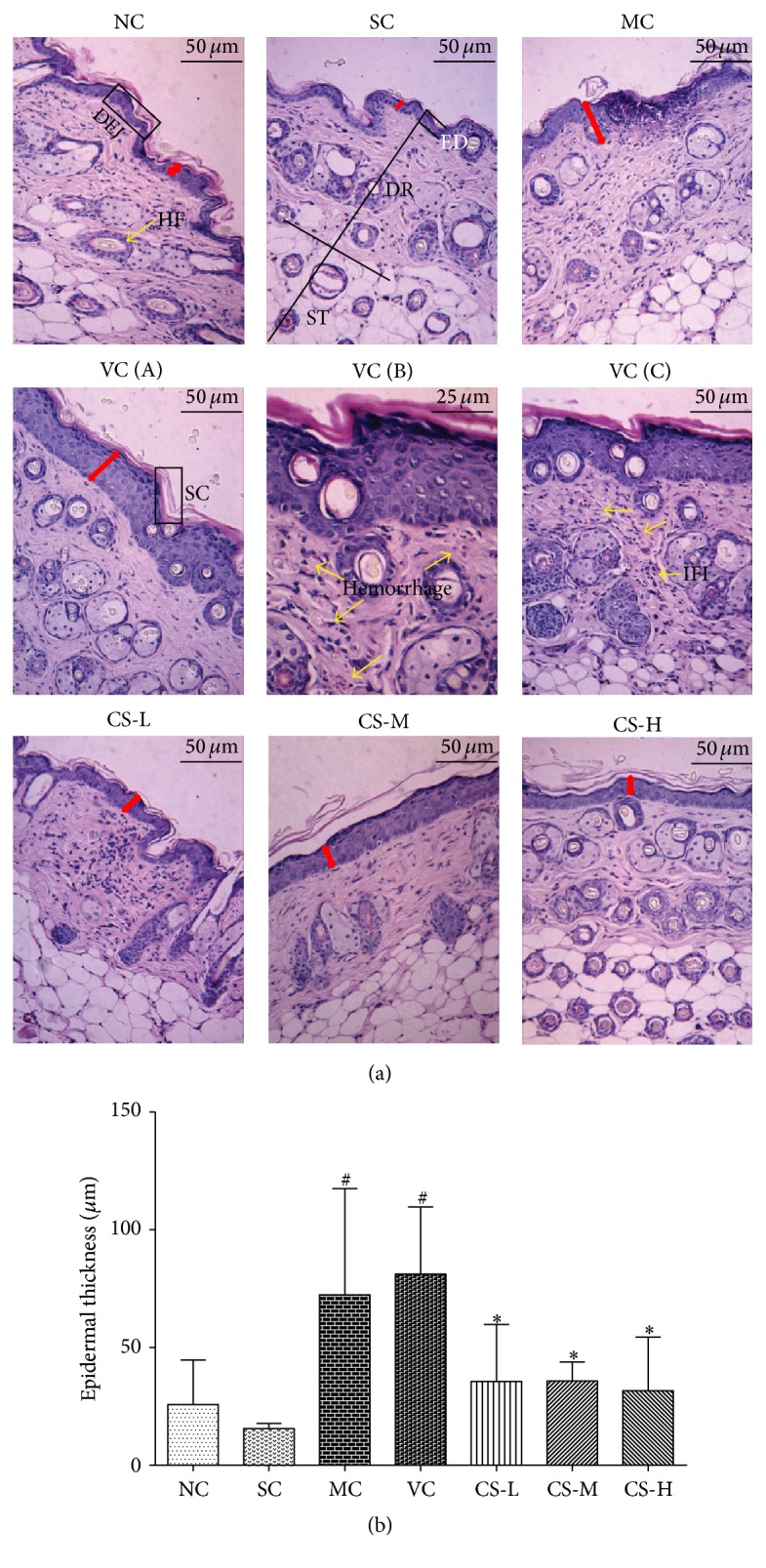
CS reduced epidermal thickness. (a) Histological images of mice skin via H&E staining. Epidermal thickness was shown via the double-headed red arrows. NC group (100x) and SC group (100x) were representing a normal structure; HF, hair follicle; DEJ, dermal-epidermal junction; ED, epidermis; DR, dermis; and ST, subcutaneous tissue. MC group and VC group were showing similar abnormal structure (100x); (A) SC, stratum corneum (100x); (B) hemorrhage (200x); (C) IF, inflammation infiltration (100x); CS-L group (100x); CS-M group (100x); and CS-H group (100x). (b) Histograms of average epidermal thickness. ^#^ *p* < 0.05 compared with the SC group; ^*∗*^ *p* < 0.05 compared with the VC group.

Epidermal thickness can be used to reflect photoaged skin, since it can result in skin roughness and winkles. As illustrated in Figure 3(b), no remarkable difference in epidermal thickness was found between the NC and SC groups as well as between the MC and VC groups, which implied that the shaving and the vehicle did not affect this indicator. Moreover, the epidermal thicknesses of mice in MC and VC groups dramatically increased after chronic UV exposure, which were 4.63 and 5.20 times of SC mice (15.62 *μ*m). However, the epidermal thickening was markedly decreased to 35.58 *μ*m, 35.85 *μ*m, and 31.68 *μ*m in the CS-L, CS-M, and CS-H groups, respectively (all *p* < 0.05 versus VC group). Besides, a result of dorsal skin-fold thickness measured by vernier caliper also supported the potential inhibitory effect of CS on skin thickening (Figure 2(c)).

### 3.4. CS Prevented UV-Induced Skin Structure Damage

**Figure 4:**
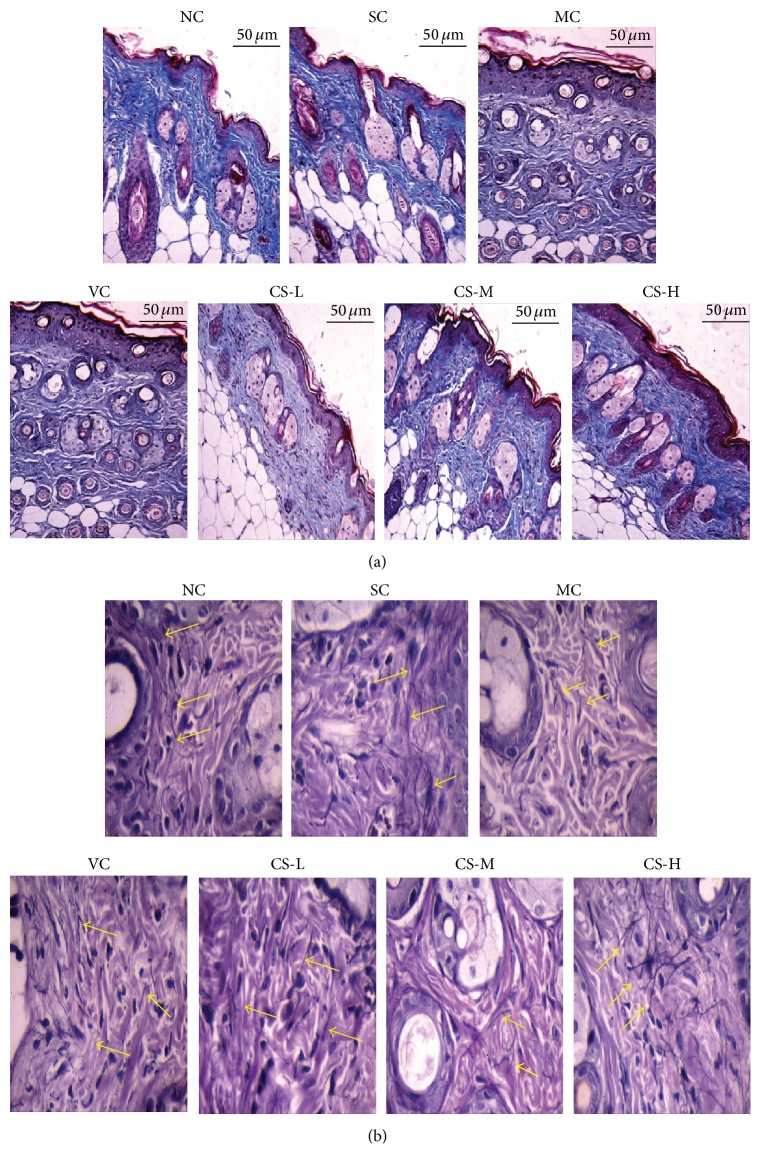
(a) Collagen fibers of mice skin via Weigert's staining (all photographs were magnified at 100x). NC and SC group presented collagen fiber bundles of high content. MC and VC groups showed disordered and scattered collagen fiber bundles. (b) Elastic fibers of mice skin via Masson's trichromatic dyeing (all photographs were magnified at 400x). NC and SC group exhibited abundant organized elastic fibers; MC and VC groups showed ruined and fractured elastic fibers.

Repeated UV irradiation led to abnormal histological alterations of the mice skin. Dorsal skins of the SC and NC groups both manifested clear and complete skin structures with thin layer of stratum corneum covering normal epidermis and wavy dermal-epidermal junction (DEJ) lines. In the superficial dermis, neither inflammatory infiltration nor hemorrhages were observed (Figure 3(a)). Hair follicles were distributed regularly and collagen bundles stained pink interweaved closely and arranged orderly (Figure 4(a)), while elastic fibers stained deep purple were slender and branched (Figure 4(b)). After ten weeks, similar histopathological features could be observed in MC and VC groups. In the epidermis, both of them showed epidermal hyperplasia and excessive keratinization as well as obviously thickened stratum corneum. Additionally, DEJ lines turned to be flattened, which could decrease the surface contact area and then cause fragile skin. In the dermis, large amounts of the collagen fiber bundles were disorganized and destructed companied with plenty fragmented elastic fibers. Moreover, skins of MC and VC groups presented hemorrhage and inflammatory infiltration in the entire dermis.

Nevertheless, CS especially at middle and high doses obviously ameliorated this structure damage caused by UV light. The skin features of CS-L group were much similar to the VC groups but presented more regular collagen bundles and less inflammatory infiltration. In CS-M and CS-H groups, skin structure damage got apparently improved (Figure 3(a)). Firstly, the epidermal thickness was significantly decreased and the DEJ lines were more curved. Secondly, abundant collagen bundles and elastic fibers were orderly displayed. Thirdly, diffused inflammation and hemorrhages were absent in and underneath the dermis in the CS-H group.

### 3.5. CS Reduced UV-Induced Oxidative Stress

**Figure 5:**
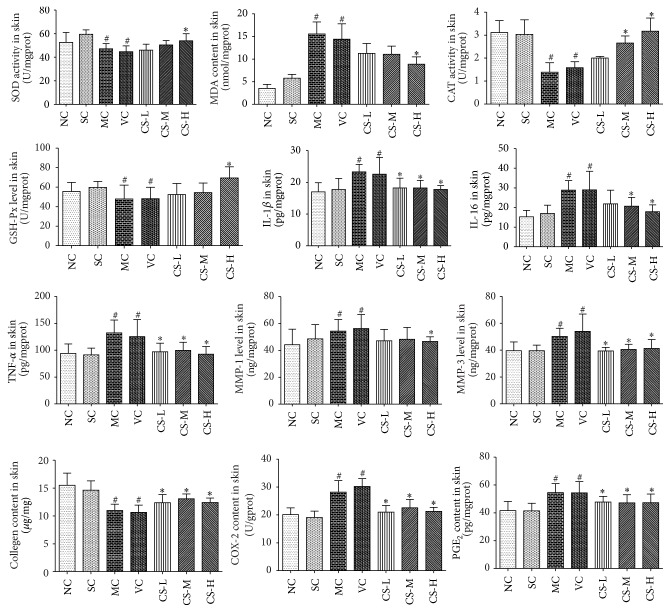
CS inhibited UV-induced oxidative stress and LPO, suppressed the generation of inflammatory cytokines, downregulated MMP-1 and MMP-3 levels, and prevented skin collagen from UV damage. Data shown are the mean values ± SD (*n* = 9). ^#^ *p* < 0.05 compared with the SC group; ^*∗*^ *p* < 0.05 compared with the VC group.

Compared to the SC group, activities of antioxidant enzymes as SOD, CAT, and GSH-Px in the MC and VC groups were obviously reduced (*p* < 0.05). However, CS at high dose could maintain the activity of SOD, CAT, and GSH-Px, which were increased by 20.71%, 68.35%, and 43.93%, respectively (all *p* < 0.05 versus VC group). These results revealed that CS enhanced the activities of antioxidant enzymes to suppress the UV-induced oxidative stress (Figure 5).

### 3.6. CS Decreased the Skin MDA Content

When compared with SC group, obvious increase of MDA level was found in MC and VC groups (*p* < 0.05 versus SC group). Nevertheless, the MDA content in the CS-H group was notably decreased by 35.11%, as compared with that in the VC group. Therefore, CS inhibited lipid oxidation by diminishing MDA and the effect of the high dose of CS was much better compared to the middle and low doses (Figure 5).

### 3.7. CS Suppressed Inflammatory Cytokines Production

Inflammatory cytokines (IL-1*β*, IL-6, and TNF-*α*) were substantially produced in the MC and VC groups (*p* < 0.05 versus SC group). However, the production of IL-1*β*, IL-6, and TNF-*α* in the CS treated groups especially in middle dose and high dose was significantly suppressed (for all *p* < 0.05 versus VC group). The results showed that CS restrained the generation of inflammatory cytokines induced by UV irradiation (Figure 5).

### 3.8. CS Inhibited COX-2 to Hinder the Synthesis of PGE_2_

The expressions of COX-2 and PGE_2_ in the MC group were much higher than these in the SC group (both *p* < 0.05 versus SC group). However, the contents of COX-2 in CS groups were reversed to about normal levels. When treated with CS, the contents of COX-2 were decreased by 30.49%, 25.40%, and 29.60%, respectively, while the PGE_2_ expressions were lower in a certain degree compared to that in the VC groups (all *p* < 0.05 versus VC group). The results showed that CS could inhibit the production of COX-2 to hinder the synthesis of PGE_2_ and thus ameliorate the skin lesions and algesia induced by UV irradiation (Figure 5).

### 3.9. CS Enhanced Skin Collagen Content in Photoaged Mice

Collagen helps skin to be firmed and flexible and plays an important role in constant cell metabolism [2]. After long time of UV irradiation, the MC and VC groups showed obvious decrease in collagen content (both *p* < 0.05 versus SC group). After being treated with CS the collagen contents were evaluated (for all *p* < 0.05 versus VC group). These results manifested that CS could protect skin collagen from UV damage. In Figure 4(a), the optical density of collagen bundles stained deep blue also confirmed the preservative effects of CS on collagen degradation (Figure 5).

### 3.10. CS Reduced Overexpression of MMPs

It is reported that MMPs (especially MMP-1 and MMP-3) are responsible for the degradation of skin collagen [5]. The contents of MMP-1 and MMP-3 in the VC group were much higher (both *p* < 0.05 versus SC group). In the CS treated groups the contents of MMP-3 were obviously decreased (all *p* < 0.05 versus VC group). What is more, CS at high dose inhibited the lift of MMP-1 (*p* < 0.05 versus VC group). These results suggested that CS could effectively reduce the overexpressions of MMP-3, as well as MMP-1, thereby preventing the UV-induced collagen degradation (Figure 5).

### 3.11. UV Absorption Spectrum and SPF of CS

**Figure 6:**
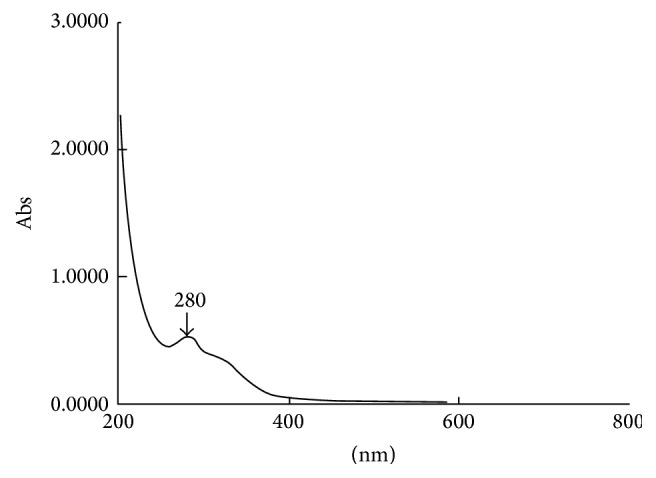
The UV absorption spectrum of CS aqueous extract (200 nm to 800 nm).

The UV absorption spectrum of CS was presented in Figure 6. The largest absorption wavelength was 280 nm. SPF values of CS were listed in Table 3, which were in positive correlation with dose. The SPF value in CS-H group was 2.05-fold to CS-M and 4.14-fold to CS-L group.

## 4. Discussion

Photoaging is a comprehensive consequence of exposure to UV irradiation, which is clinically characterized with sagging, coarseness, wrinkling, erythema, and dyspigmentation. Consistent with the previous studies, UV irradiation visibly accelerated wrinkle formation and increased skin thickness in our study. However, CS had a tendency to alleviate the apparent lesions and slow down the progress of photoaging, confirmed by the fact that the formation of wrinkles and the skin-fold thickness in the CS groups were apparently lower than those in the VC group. The SPF values also confirmed the photoprotection effects of CS, which were in a dose-dependent manner. Results of HPLC-ESI-MS analysis showed that compounds as caffeic acid [24], ferulic acid [25], ursolic acid [26], p-hydroxybenzoic acid [27], rosmarinic acid [28], and protocatechuic acid [29] existed in CS. These compounds had been reported to exhibit protective effect against UV irradiation* in vitro *via reducing oxidative stress. These active substances made the foundation of photoprotective ability of CS.

Histological changes were characterized with increased thickness of stratum corneum and excessively keratinized epidermis after exposure to UV. In the dermis of photoaged skin, the collagen fiber bundles were tangled and destructed companied with elastic fibers dramatically degraded. The primary mechanism of these abnormal histological alterations was overproduction of MMPs, such as MMP-1 and MMP-3. MMP-1 (interstitial collagenase-1) breaks down fibrillar collagens and elastin, while MMP-3 (stromelysin-1) degrades elastic fibers and other connective components, therefore resulting in a loss of skin's ability to resist stretching [1]. In this study, we found that CS suppressed the increase of MMP-1 and MMP-3 production and promoted the density of collagen and elastic fibers. These results indicated that CS can be used as MMPs inhibition to remodel extracellular matrix structures in the tissue, thereby alleviating UV-induced skin damage.

It has been reported that excessive MMPs resulted from resultant accumulation of ROS and inflammation responses. The protection mechanism against ROS damage is antioxidant defense system [30]. Intrinsic antioxidant enzymes could scavenge ROS and protect skin cells. SOD accelerates the reduction of O_2_^−^ into O_2_ and H_2_O_2_, and CAT catalyzes H_2_O_2_ into O_2_ and H_2_O [30]. SOD cooperated with CAT in eliminating O_2_ initiated ROS. In addition, GSH-Px also breaks down H_2_O_2_ with the substrate glutathione [30]. Although there are efficient antioxidant systems in skin, the excessive ROS induced by UV exposure exceeds their capacity. Hsu et al. [10] had determined ROS scavenging and Trolox equivalent antioxidant capacity (TEAC) as well as Oxygen Radical Absorbance Capacity (ORAC) assay of CS water extract* in vitro*. The results of that study fully proved the ROS scavenging potential and antioxidant property of CS. In this study, CS especially at high dose could protect antioxidant enzymes and reduce oxidative stress. These results revealed that CS possessed antiphotoaging effect on the foundation of scavenging free radical and enhancing antioxidant activities. Such antioxidant efficacy of CS* in vivo* was owed to phenolic acids in CS such as rosmarinic acid, caffeic acid, and danshensu. These phenolic acids not only exhibited both free radical scavenging activities to defend body's antioxidant mechanism but also stimulated tyrosinase to expedite melanin production [24, 28, 31, 32].

What is more, lipid peroxidation (LPO) is initiated by ROS on polyunsaturated fatty acids and aggravates LPO in membranes and cellular components and subsequently causes cell death and accumulation of abnormal proteins and cellular debris, finally leading to serious skin pathologies [33]. LPO can be further decomposed to a number of reactive aldehyde species such as MDA [34]. Therefore, MDA is usually used as a parameter to quantify LPO. However, CS could downregulate the accumulation of MDA in mice skin. Protocatechuic acid and rosmarinic acid in CS have been reported to have antilipid peroxidative properties through modulating of cellular redox status with the upregulated expression of antioxidant enzymes, including heme oxygenase-1, SOD, and CAT. Therefore, CS could protect the UV-induced skin damage by depressing LPO [31, 35].

Furthermore, a characteristic response of keratinocytes to chronic UV irradiation is the oxidation of the arachidonic acid in cell membranes, which is catalyzed by the enzyme COX-2. COX-2 can result in the formation of oxidation products such as PGE_2_, which are additional mediators of inflammation reaction. The expression of COX-2 has been documented in inflammation response, while intradermal PGE_2_ is hyperalgesic in the peripheral nervous system. PGE_2_ acts as a potent vasodilator and synergistically with other mediators increases vascular permeability and other inflammatory markers such as erythema, edema, and hyperplastic epithelial responses [6]. Expression of COX-2 is upregulated by ROS to stimulate the inflammation process. However, CS significantly reduced the contents of COX-2 and PGE_2_ in mice skins. It could be due to the chemical compounds in CS such as caffeic acid phenethyl ester [36], protocatechuic acid [37], rosmarinic acid [28], and baicalein [38] to confer anti-inflammatory properties.

Exposure to UV light usually motivates inflammation. The regulatory mechanisms include releasing of upstream proinflammatory cytokines such as TNF-*α* from keratinocytes. UV irradiation stimulates TNF-*α* synthesis and releases it into blood stream, which activates downstream cytokines such as IL-3, IL-1*β*, and IL-6. These inflammatory factors mediate the growth of keratinocytes in epidermis and lead to epidermal hyperplasia [1]. Consistent with the previous research, the contents of IL-1*β*, IL-6, and TNF-*α* were sharply lifted after exposure to UV in our study. However, when treated with CS, IL-1*β*, IL-6, and TNF-*α* contents were remarkably reduced to about normal level. The inflammatory infiltration could not be observed in the H&E sections of CS-H group, which was proved by smooth skin appearance (Figure 2(a)). Combined with results of HPLC-ESI-MS/MS analysis, chemical components in CS such as baicalein and protocatechuic aldehyde were crucial for its distinguished anti-inflammatory activity through inhibition of COX-2 gene expression by blockade of C/EBPbeta DNA binding activity [38] and the TNF-*α*-activated NF-*κ*B and AP-1 DNA binding activities [39].

In conclusion, our research for the first time applied CS to the mice skin and demonstrated that CS could postpone exogenous senescence via defending antioxidant activities and suppressing inflammatory response. It indicated that CS is a potential agent for antiphotoaging cosmetics.

## Figures and Tables

**Table 1:** Grading scales for evaluation of photoaging.

Grade	Evaluation criteria
0	No wrinkles; fine striations running the length of the body
1	Fine striations
2	A few shallow wrinkles; disappearance of all fine striations
3	Shallow wrinkles across the dorsal skin
4	Deep and coarse wrinkles with laxity
5	Increased deep wrinkles
6	Severe wrinkles; development of tumors or lesions

**Table 2:** HPLC-ESI-MS analysis of CS aqueous extract.

Number	RT (time)	Molecular weight	Parent ion (*m*/*z*)		Product ion (*m*/*z*)	Compounds^a^
			[M + H]^+^	[M − H]^−^		
1	4.4	374.34	375.1	—	231, 213, 114	Methyl rosmarinate
2	5.3	226	—	225.1	223, 179, 161, 87	Ethyl 3,4-dihydroxyphenyllactate
3	5.7	270.24	—	269.1	109, 88, 61	Baicalein
4	6.7	198.17	—	197.0	135, 123, 109, 73	Danshensu
5	12.8	154.12	—	153.0	119, 108, 91	Protocatechuic acid
6	21.5	138.12	—	137.0	93, 65	p-Hydroxybenzoic acid
7	22.4	360.31	—	359.1	197, 179, 161, 135	Rosmarinic acid
8	24.8	180.15	—	179.0	136, 135, 134	Caffeic acid
9	40.5	194.18	—	193.1	165, 150, 78	Ferulic acid
10	45.4	472.69	473.36	—	464, 416, 408, 360	2*α*-Hydroxy-ursolic acid

^a^Analyzed by HPLC-ESI-MS analysis both in positive and in negative ionization modes.

**Table 3:** SPF values of CS aqueous extract at different doses.

Doses of CS (g/mL)	SPF values
0.9	3.92 ± 0.01
1.8	7.90 ± 0.02
3.6	16.21 ± 0.03
